# Early Gut Microbiota Perturbations Following Intrapartum Antibiotic Prophylaxis to Prevent Group B Streptococcal Disease

**DOI:** 10.1371/journal.pone.0157527

**Published:** 2016-06-22

**Authors:** Giuseppe Mazzola, Kiera Murphy, R. Paul Ross, Diana Di Gioia, Bruno Biavati, Luigi T. Corvaglia, Giacomo Faldella, Catherine Stanton

**Affiliations:** 1 Department of Agricultural Sciences, University of Bologna, Bologna, Italy; 2 Food Biosciences Department, Teagasc Food Research Centre, Fermoy, Cork, Ireland; 3 School of Microbiology, University College Cork, Cork, Ireland; 4 Alimentary Pharmabiotic Centre, University College Cork, Cork, Ireland; 5 Neonatal Intensive Care Unit, S. Orsola Malpighi Hospital, Bologna, Italy; University of Illinois at Urbana-Champaign, UNITED STATES

## Abstract

The faecal microbiota composition of infants born to mothers receiving intrapartum antibiotic prophylaxis with ampicillin against group B *Streptococcus* was compared with that of control infants, at day 7 and 30 of life. Recruited newborns were both exclusive breastfed and mixed fed, in order to also study the effect of dietary factors on the microbiota composition. Massive parallel sequencing of the V3-V4 region of the 16S rRNA gene and qPCR analysis were performed. Antibiotic prophylaxis caused the most marked changes on the microbiota in breastfed infants, mainly resulting in a higher relative abundance of Enterobacteriaceae, compared with control infants (52% vs. 14%, p = 0.044) and mixed-fed infants (52% vs. 16%, p = 0.13 NS) at day 7 and in a lower bacterial diversity compared to mixed-fed infants and controls. Bifidobacteria were also particularly vulnerable and abundances were reduced in breastfed (p = 0.001) and mixed-fed antibiotic treated groups compared to non-treated groups. Reductions in bifidobacteria in antibiotic treated infants were also confirmed by qPCR. By day 30, the bifidobacterial population recovered and abundances significantly increased in both breastfed (p = 0.025) and mixed-fed (p = 0.013) antibiotic treated groups, whereas Enterobacteriaceae abundances remained highest in the breastfed antibiotic treated group (44%), compared with control infants (16%) and mixed-fed antibiotic treated group (28%). This study has therefore demonstrated the short term consequences of maternal intrapartum antibiotic prophylaxis on the infant faecal microbial population, particularly in that of breastfed infants.

## Introduction

Group B *Streptococcus* (GBS), a gram-positive commensal bacterium residing in the gastrointestinal and genitourinary tract of 10–30% of pregnant women, is the primary cause of early-onset bacterial sepsis in newborns, which represents a major cause of neonatal morbidity and mortality [1]. The neonate can be exposed to GBS in utero, where the organism can reach the amniotic fluid through ruptured membranes, and also during passage through the birth canal. In addition to maternal colonization with GBS, other factors that increase the risk for early-onset infection include prematurity, rupture of membranes or an intrapartum temperature >37.5°C [2]. The introduction of universal GBS screening of all pregnant women in many countries and subsequent intrapartum antibiotic prophylaxis (IAP) in GBS-positive women has seen a significant reduction in early-onset GBS infection from 1.7 to 0.25 cases per 1,000 births in the United States [3].

While the use of IAP is clearly advantageous in the prevention of GBS infection, there is uncertainty about its impact on the development of the infant gut microbiota. The first microbial population the neonate encounters is the maternal vaginal and faecal microbiota, followed by microorganisms from the external environment [4,5]. The early colonizers are facultative anaerobes [6], whereas the establishment of a reducing environment permits the growth of strict anaerobes, such as *Bifidobacterium*, *Bacteroides* and *Clostridium*. Microbial diversity and richness continues to increase until the cessation of weaning when the microbiota becomes similar to that of the adult [7–9]. It is well known that colonization in the early days after birth is influenced by several factors including the mode of delivery (vaginal vs. caesarean section), feeding choice (breast vs. formula feeding), gestational age (preterm vs. full-term), hospitalization after birth and antibiotic use [10,11][12].

There are relatively few reports focusing on the possible effect that maternal IAP treatment may have on the microbiota composition in infants [13,14]. The techniques used in previous studies, such as plate counts, qPCR and PCR-DGGE, did not allow a complete analysis of the entire faecal microbiota composition. Thus, the objective of this study was to use whole genome sequencing in combination with qPCR to thoroughly examine the impact of maternal IAP on the faecal microbiota composition during the first month of life. In addition, the effect of dietary factors, i.e. exclusive breast-feeding versus mixed feeding, was also investigated.

## Materials and Methods

### Study design and samples collection

Ethical approval was received by the Comitato Etico Indipendente of S. Orsola-Malpighi Hospital in Bologna (protocol 12/2013/U/Oss approved on March 12,2013). Twenty six infants born at the Neonatal Intensive Care Unit, S. Orsola-Malpighi Hospital were recruited and belonged to one of four groups: 1) BF-C, Breast-fed (BF) infants born to GBS negative mothers, not receiving IAP before/at delivery (control); 2) BF-IAP, BF infants born to GBS positive, IAP treated mothers; 3) MF-C, Mixed-fed (MF) infants born to GBS negative mothers (control); 4) MF-IAP, MF infants born to GBS positive, IAP treated mothers (Table 1). Infants were born at term by vaginal delivery, weighed between 2.5 and 4.0 kg at birth and did not receive any perinatal antibiotic or probiotic/prebiotic treatment. Written informed consent was obtained from parent/legal guardian for participation in the study. Women who screened positive for GBS by rectovaginal culture at 35–37 week gestational age were treated with intravenous ampicillin (Amplital^®^) at labour every four hours until delivery (first dose 2g, following doses 1g each to a maximum of 4g).

**Table 1:** Characteristics of infants in the four groups of this study.

	BF-IAP	BF-C	MF-IAP	MF-C
**No. of subjects**	7	7	6	6
**Gender** (Female/Male)	2/5	2/5	4/2	5/1
**Mode of Delivery**	SVD	SVD	SVD	SVD

BF = exclusive breast-feeding

MF = a combination of formula (at least 50%) and breast milk feeding

SVD = Spontaneous vaginal delivery.

Faecal samples were collected from each infant during follow up visits at day 7 and 30 of life. At each visit, information on weight gain, clinical conditions, feeding, and on-going treatments (i.e. use of prebiotics, probiotics, antibiotics), were collected from the infants' parents. After collection, faecal samples were immediately frozen at -80°C, until processing for bacterial DNA extraction.

### DNA extraction from infant faecal samples

Total bacterial DNA was extracted from 200 mg of stool using the QIAamp DNA Stool Mini Kit (Qiagen, West Sussex, UK), according to the manufacturer’s instructions with an additional incubation at 95°C for 10 min with lysis buffer to improve the efficiency of bacterial cell rupture for Gram-positive bacteria. Extracted DNA was stored at -80°C.

### Preparation of 16S V3 and V4 rRNA amplicons for Illumina MiSeq sequencing

Extracted DNA was processed to amplify and sequence the V3-V4 region of the 16S rRNA gene [15]. These amplicons, approximately 550 bp in length, were generated using the forward primer, 5’-TCGTCGGCAGCGTCAGATGTGTATAAGAGACAGCCTACGGGNGGCWGCAG-3’ and the reverse primer, 5’-GTCTCGTGGGCTCGGAGATGTGTATAAGAGACAGGACTACHVGGGTATCTAATCC-3’. Each 25 μl PCR reaction contained 12.5 μl of HiFi HotStart ReadyMix (KAPA Biosystems, Woburn, MA), 5 μl of each primer (0.2 μM) and microbial DNA (5 ng/μl). PCR amplification was performed using the following program: heated lid at 110°C, 95°C for 3 min followed by 25 cycles at 95°C for 30 s, 55°C for 30 s, 72°C for 30 s, followed by a final elongation step at 72°C for 5 min. PCR products were cleaned using the AMPure XP purification system (Beckman Coulter, UK). Illumina sequencing adapters and dual-index barcodes were added to amplicons using the Nextera XT index kit (Illumina, San Diego, CA). The following program was utilized for the second PCR amplification: 95°C for 3 min followed by 8 cycles of 95°C for 30 s, 55°C for 30 s and 72°C for 30 s and a final elongation at 72°C for 5 min. Amplicons were cleaned as outlined and quantified using the Qubit^®^ 2.0 Fluorometer (Invitrogen, Life Technologies, CA, USA). The quantified libraries were pooled equimolar to 4 nM prior to denaturation with 0.2N NaOH, further dilution with hybridization buffer to 4pM and combined with 10% (v/v) denatured 4pM PhiX. Samples were sequenced on the Illumina MiSeq platform at the Teagasc Food Research Centre facility using a 2x300 nucleotide paired-end protocol.

### Bioinformatic and statistical analysis

Raw sequence reads were assembled in to 300bp paired-ends using FLASH [16]. Reads were further processed using the QIIME suite of tools, version 1.8.0 [17], including quality filtering based on a quality score of >25 and removal of mismatched barcodes and sequences below length thresholds. Denoising, chimera detection, and operational taxonomic unit (OTU) grouping were performed using USEARCH v7 [17]. Taxonomic ranks were assigned to each sequence by alignment of OTUs using PyNAST [18] to the SILVA SSURef database, release 111. Alpha and beta diversities were generated in QIIME using a rarefied OTU table and calculated based on weighted and unweighted Unifrac distance matrices [19]. Principal coordinate analysis (PCoA) plots were visualised using EMPeror v0.9.3-dev [20]. MiniTab release 17 (MiniTab Ltd. Coventry, UK) was used to perform statistical analysis. The nonparametric Mann-Whitney analysis or Wilcoxon signed rank test were used to evaluate sample differences in a pairwise manner. A corrected p-value of less than 0.05 was considered significantly different.

### Absolute quantification of total bacteria and bifidobacteria using quantitative PCR (qPCR)

Total bacteria were quantified as described by Guo et al. (2008) [21] and *Bifidobacterium breve* DSM 20213 as the reference strain. *Bifidobacterium* spp. were quantified as already described [13]. Specificity for target organisms was evaluated using the BLASTN algorithm [22] and melting curve analysis. Standard curves were established using 10^2^ to 10^6^ copies 16S rRNA/μl. qPCR reactions were performed in triplicate in a StepOne RealTime PCR System (Applied Biosystems, Foster City, CA).

## Results

### Fecal microbiota composition of BF-IAP and BF-C fecal infants

The V3-V4 region of bacterial 16S rRNA gene was amplified and subjected to massive parallel sequencing. A total of 11,109,005 raw sequences were obtained with an average of 217,823 reads per sample and were classified into 427 OTUs.

**Fig 1:**
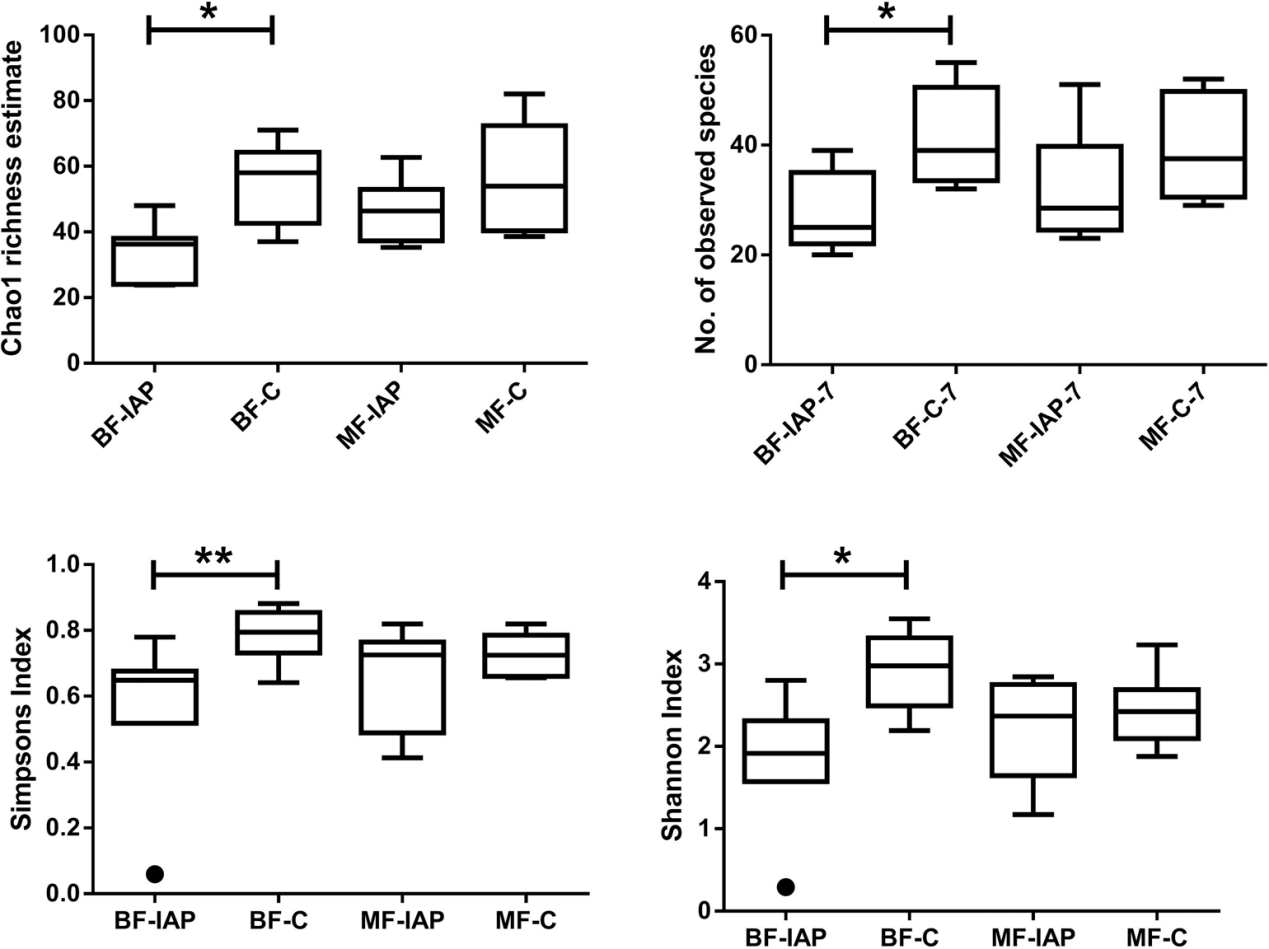
Estimates of alpha diversity for BF-IAP, BF-C, MF-IAP and MF-C samples at day 7. One outlier has been identified in BF-IAP group regarding the evaluation of Simpson and Shannon indices, indicated with a circle.

Alpha diversity analysis based on Chao1 (p = 0.0122), Simpson (p = 0.035), Shannon (p = 0.0082) and observed species (p = 0.021) indices revealed significantly lower diversity in BF-IAP infants compared with BF-C at day 7 (Fig 1). At day 30, the Chao1 index and observed species increased in the BF-IAP infants, although this was not a significant increase, and the Simpson and Shannon indices remained largely unchanged (S1 Fig).

**Fig 2:**
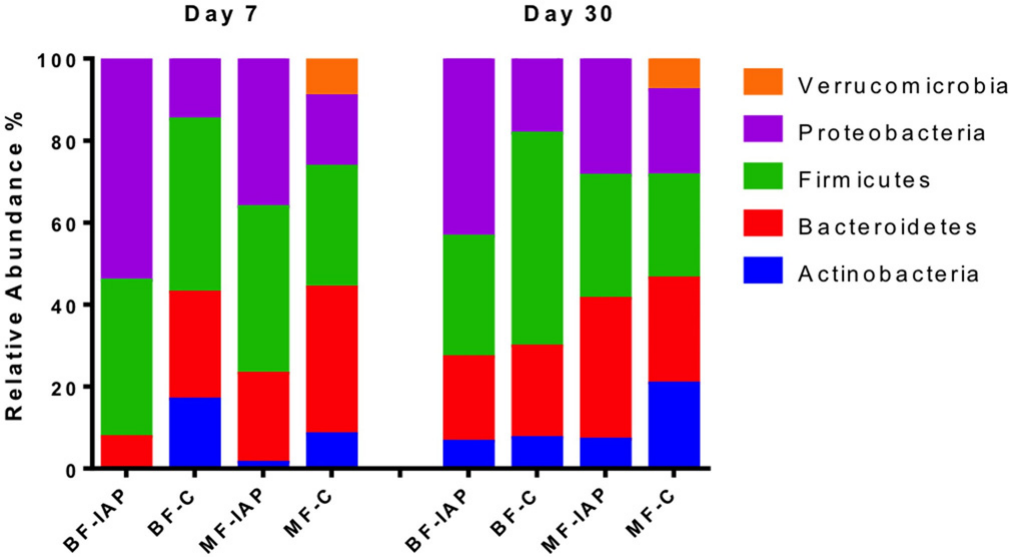
Relative abundances of bacterial phyla in BF-IAP, BF-C, MF-IAP and MF-C faecal samples.

**Fig 3:**
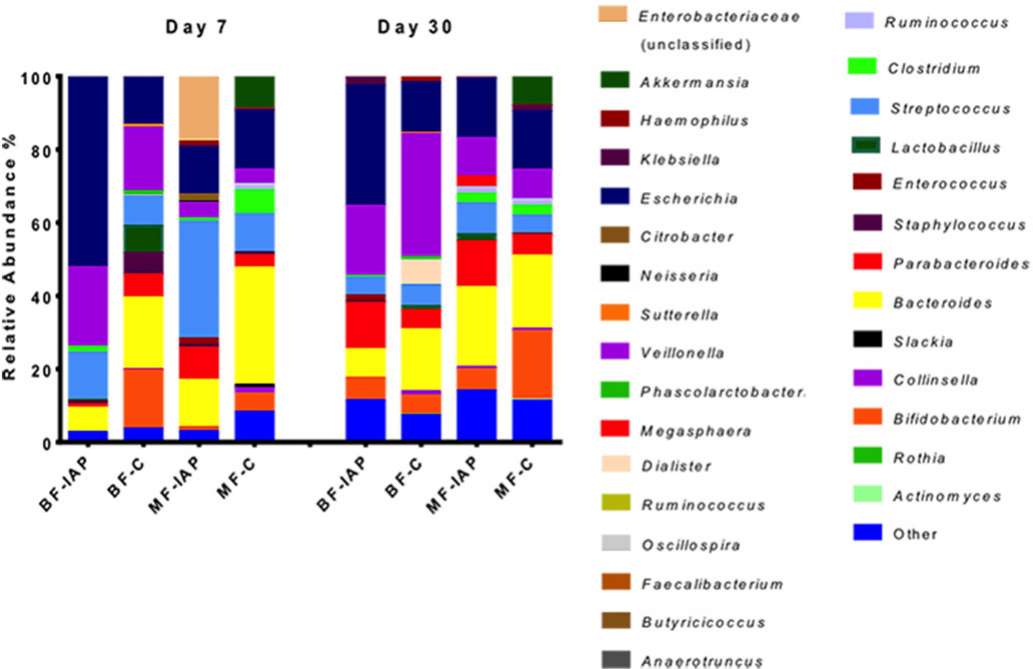
Relative abundances of bacterial genera in BF-IAP, BF-C, MF-IAP and MF-C faecal samples. Other category contains genera present at <1% of relative abundance.

At phylum level, Actinobacteria were not detected in the BF-IAP infants and were present at 17% in control infants (p<0.001). BF-IAP infants had significantly higher abundances of Proteobacteria (P<0.062) at day 7 compared to BF-C infants (Fig 2). BF-IAP infants were dominated by genera belonging to the Enterobacteriaceae family (p = 0.044), particularly *Escherichia* which accounted for 52% of the total relative abundance, compared with 14% in the BF-C group (Fig 3). Bifidobacteria were not detected in any of the BF-IAP infants at day 7; in contrast, *Bifidobacterium* represented 16% of the relative abundance of the microbiota from BF-C infants (p = 0.001). BF-C infants also had higher levels of *Bacteroides*, 20% compared with 7% in BF-IAP, although not statistically significant (p = 0.078) (Fig 3). By day 30, bifidobacteria numbers appeared to have recovered in the BF-IAP group and now account for 6% of the relative abundance (p = 0.025) in both BF groups. Enterobacteriaceae continue to dominate in BF-IAP infants, being detected at 44%, in contrast with 16% in BF-C. Additionally, at day 30 significantly higher levels of the Veillonellaceae family were detected in BF-C infants compared with BF-IAP (p = 0.035) (S2 Fig).

**Fig 4:**
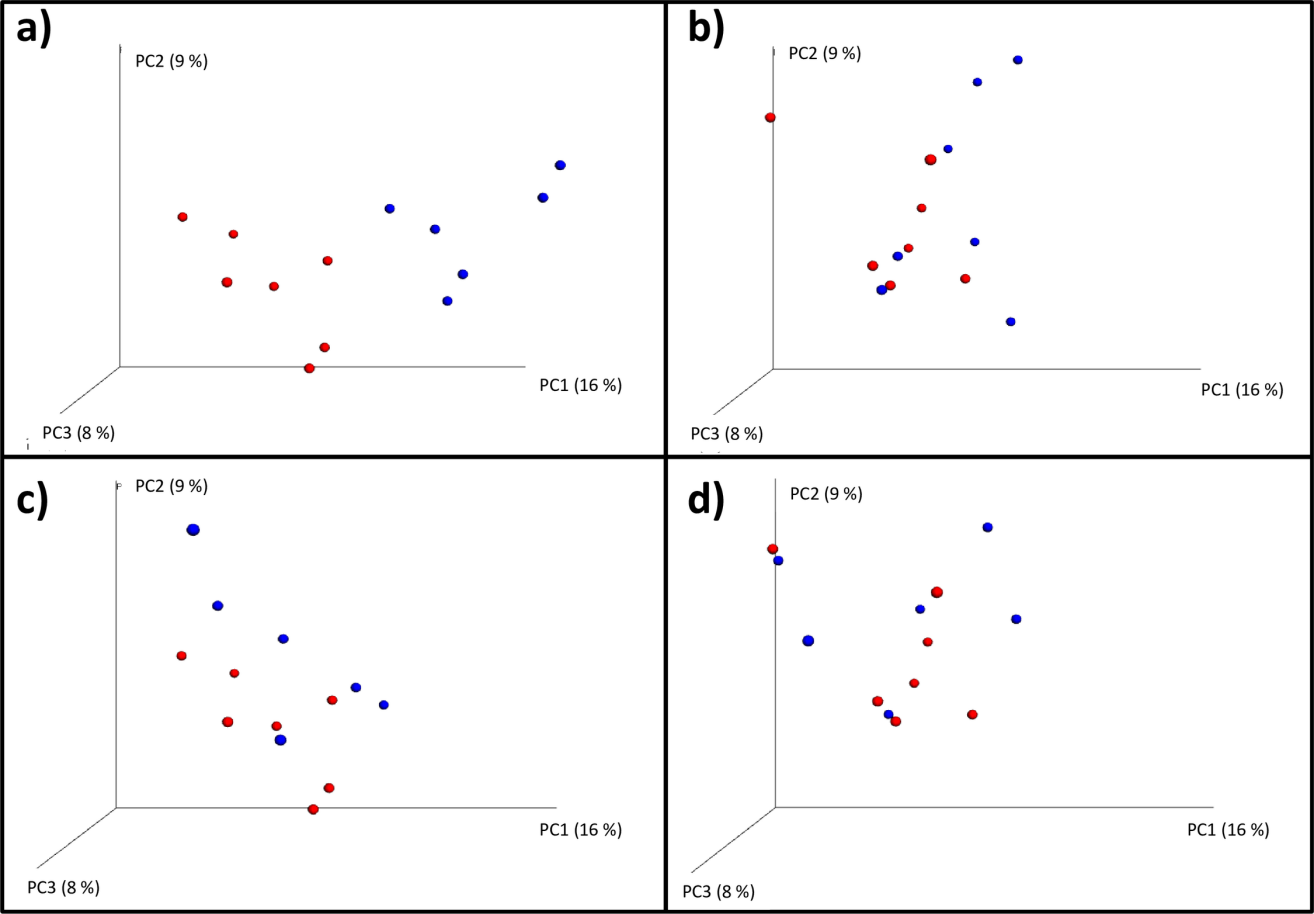
Principal coordinates analysis (PCoA) of unweighted UniFrac distances. Percent of dataset variability explained by each principal coordinate is shown in brackets in axis titles. Percent of dataset variability explained by each principal coordinate is shown in brackets in axis titles a) BF-IAP infants at day 7 in red, BF-C infants at day 7 in blue. b) BF-IAP infants at day 30 in red, BF-C infants at day 30 in blue. c) BF-IAP infants at day 7 in red, MF-IAP infants at day 7 in blue. d) BF-IAP infants at day 30 in red, MF-IAP infants at day 30 in blue.

Principal coordinate analysis (PCoA) plots constructed using unweighted UniFrac distance matrices shows clear separation of BF-IAP samples from those of the BF-C infants at day 7, no clear separation was observed at day 30, suggesting that microbial communities became more uniform over time (Fig 4).

### Fecal microbiota composition of MF-IAP and MF-C infants

No significant differences in diversity at day 7 was observed within mixed-fed infants, although Chao1, Shannon and observed species indices were highest in the MF-C infants (Fig 1). By day 30, alpha diversity was similar in both MF groups. MF-IAP infants contained higher abundances of Proteobacteria, 37%, and Firmicutes, 41%, compared with MF-C infants, 17% and 29%, respectively, at day 7 (Fig 2). Actinobacteria (8%) and Bacteroidetes (36%) were highest in the MF-C group compared with 1% and 21% in MF-IAP infants. MF-IAP infants contained high abundances of organisms belonging to family Enterobacteriaceae, 35%, and *Streptococcus*, 32% (Fig 3), compared with 17% and 10% in the control group. MF-C infants had higher levels of *Bacteroides*, 32%, and *Bifidobacterium*, 5%, compared with 13% and 1%, respectively, in MF-IAP infants (Fig 3).

At day 30, Bacteroidetes were the dominant phylum in both groups, representing 26% in control and 34% in IAP treated infants. Actinobacteria levels increased in the MF-IAP infants to 7% and Firmicutes and Proteobacteria reduced to 30% and 28% respectively. At genus level, the microbiota composition was more uniform than that at day 7. Members of the Enterobacteriaceae family fall to 28% and *Streptococcus* was significantly reduced to 8% (p = 0.042). The Lachnospiraceae family, absent at day 7 in IAP treated infants, was detected at 4% at day 30. Bifidobacteria significantly increased in MF-IAP infants from 0% at day 7 to 6% at day 30, (p = 0.013) and remain highest in MF-C, 19%.

Moreover, Fig 3 shows that *Veillonella* is affected by the antibiotic treatment, as it does not increase in the BF-IAP group between 7 and 30 days, whereas a strong increase is shown within BF-C samples at the same sampling times (40%) and in BF-C samples with respect to BF-IAP at 30 days (43%).

Principal coordinate analysis showed no clustering of samples from either MF-IAP or MF-C groups at day 7 or 30 (data not shown).

### Effects of dietary factors: comparison between BF-IAP and MF-IAP infants

The gut microbiota of BF-IAP infants had lower diversity compared with the MF-IAP group based on the Chao1, Simpson, Shannon and observed species indices, although not significant (Fig 1). At day 30, diversity increased in the BF-IAP group although diversity indices remain highest in the MF-IAP infants (S1 Fig).

At day 7, Actinobacteria were detected at 1% in MF-IAP and were absent in the BF-IAP infants. The BF-IAP communities were dominated by Proteobacteria (54%) compared with 36% in MF-IAP, while MF-IAP had higher Bacteroidetes, 22%, compared with 7% in BF-IAP (Fig 2). Firmicutes were similar in both groups (about 40%). Enterobacteriaceae were highest in the BF-IAP group, in particular *Escherichia* were detected at 52% in BF-IAP compared with 14% in MF-IAP. BF-IAP infants were also found to have higher *Veillonella*, 22%, compared with 4% in the MF-IAP group. *Streptococcus* were abundant in the MF-IAP infants 32% versus 12% in BF-IAP and *Bacteroides* were also highest in the MF-IAP group, 13%, compared with 6% in BF-IAP (Fig 3).

At day 30, Actinobacteria and Firmicutes abundances were similar in both groups, 6% and 29% in BF-IAP, and 7% and 30% in MF-IAP. Proteobacteria remained highest in BF-IAP, 44%, compared with 28% in MF-IAP. Bacteroidetes increased in the BF-IAP to 21% and remain highest in MF-IAP 34%. BF-IAP infants were found to have higher *Veillonella* then MF-IAP, 19% and 10%, respectively, and *Escherichia*, 33% and 16% (p = 0.04), respectively. MF-IAP infants had higher *Bacteroides*, 22%, compared with 7% in the BF-IAP group. Bifidobacteria were detected in both groups at 6%, showing a significant change from day 7 (BF-IAP p = 0.025, MF-IAP p = 0.013). PCoA plots at day 7 show a separation of samples from BF-IAP and MF-IAP, whereas no clear separation was observed at day 30 (Fig 4).

### Absolute quantification of total bacteria and bifidobacteria via q-PCR

**Fig 5:**
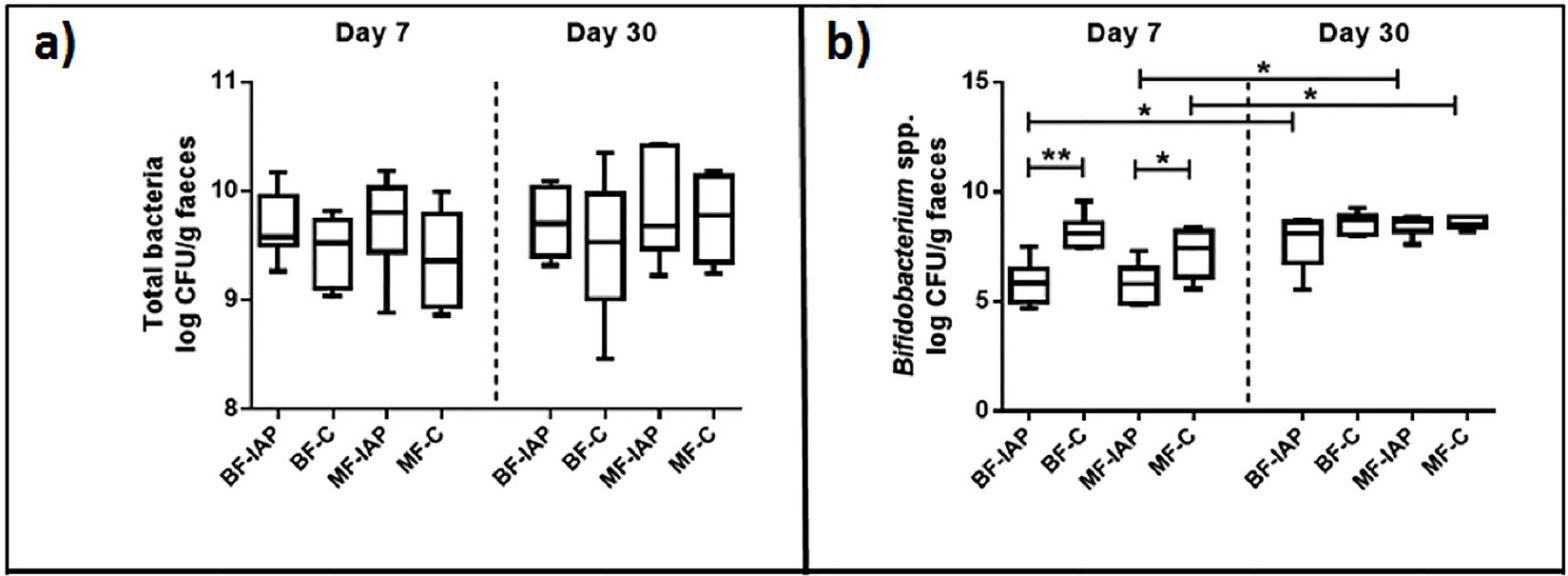
Box plots showing qPCR analysis of selected microbial groups expressed in log CFU per gram of faecal sample. Boxes show the median, 25th and 75th percentiles. Corrected p-values of < 0.05 and 0.01 are denoted by * and **, respectively.

Fecal samples at day 7 and 30 of life from each group, BF-IAP, BF-C, MF-IAP and MF-C, were subjected to qPCR analyses targeting total bacteria and bifidobacteria. Total bacteria numbers were similar across the four groups, ranging between 9.38 to 9.71 at day 7 and 9.53 to 9.83 log CFU/g at day 30, with no significant differences between groups observed (Fig 5A).

In the analysis of *Bifidobacterium* spp., numbers were observed to be significantly lower in BF-IAP infants, 5.86 log CFU/g, compared with BF-C, 8.16 log CFU/g, at day 7 (p = 0.005) (Fig 5B). Similar observations were made for MF-IAP infants, 5.81 log CFU/g, compared with 7.19 log CFU/g in MF-C infants (p = 0.03). At day 30, a significant increase was observed in both BF-IAP and MF-IAP infants to 7.72 and 8.50 log CFU/g respectively (p = 0.035 and p = 0.036). Numbers remain higher in BF-C infants, 8.62 log CFU/g, compared to 7.72 log CFU/g in BF-IAP at day 30 but is no longer a significant difference. Numbers have significantly increased in MF-C to 8.55 log CFU/g (p = 0.028) and are similar to that of MF-IAP infants, 8.50 log CFU/g.

## Discussion

The use of antibiotics in early life is known to alter the commensal gut microbiota [7,23,24], however, only few studies have focused on the effects of maternal intrapartum antibiotic administration on the infant faecal microbiota. The investigators have previously found that maternal IAP is responsible for a significant decrease in *Bifidobacterium* counts in exclusively breast-fed, vaginally delivered infants at 7 days of life using qPCR [14]. The aim of the present study, therefore, is to study microbial differences in further detail by analysing, for the first time, the entire faecal microbiota composition in infants receiving or not receiving maternal IAP by massive parallel sequencing and to see possible implications of antibiotic use during labour. In addition, the microbiota of the same infants was analyzed at one month of age and the influence of dietary factors was explored by including in the study mixed-fed newborns.

The analysis presented here shows that the faecal microbiota of BF-IAP infants was significantly reduced in diversity and richness at day 7 with respect to BF-C infants. The most marked differences were found within the Enterobacteriaceae family and the *Bifidobacterium* genus, with numbers significantly higher and lower, respectively, in the BF-IAP group. These observations are consistent with Edwards (2002) [25], whose study showed that IAP increased exposure of neonates to ampicillin-resistant Enterobacteriaceae and Tanaka et al. (2009) [26], who established an inverse relation between Enterobacteriaceae and *Bifidobacterium* counts following antibiotic exposure in the early post-natal period. The bifidobacterial population appear to have recovered in the BF-IAP day 30 samples, with a significant increase and relative abundances quite similar to BF-C. On the contrary, Enterobacteriaceae remain highest in BF-IAP infants. Massive parallel sequencing showed significant differences between MF-IAP and MF-C infants at day 7, in particular regarding Actinobacteria and Bacteroides, which were partially recovered at day 30. Recovery within the *Bifidobacterium* genus was also supported by qPCR analysis. *Veillonella* members also appear to be strongly affected by IAP and this may have consequences in the future development of the microbiota, considering that *Veillonella* is known to be crucial for the gut microbiota development at an early stage [27] and it is involved in the metabolism of lactic acid to short chain fatty acids, which are beneficial for colonocyte health [28].

Dietary factors had a significant impact on the fecal microbiota composition, as shown by the lower bacterial diversity and the lower species richness in the BF-IAP infants compared to MF-IAP and the differences in the dominant microbial groups. A potential explanation is that IAP exposure could alter the composition of the breast-milk microbiome which is known to be rich in bifidobacteria and, consequently, microbial species transmission to the infant is modified [29]. On the other hand, recent studies have outlined preliminary evidences of bifidobacteria transmission from breast milk to the newborn [30]. Moreover, breastfed infants may absorb antibiotic molecules directly from breast milk leading to modifications in some microbial groups of the intestinal microbiota, as already shown in the literature [27,31]. Our study also outlines that *Bifdobacterium* abundances significantly increased in both BF and MF groups from day 30.

As previously outlined, this work has shown that one of the main implication of antibiotic use during labour is the increase in Enterobacteriaceae which may cause infections and gut problems in neonates [32]. Therefore, intervention strategies to be applied in the early stage of life in order to re-equilibrate the gut microbiota composition are envisaged. The use of probiotics, in particular bifidobacteria, can be one of them, as also shown by studies performed by several authors [33–35].

In conclusion, massive sequencing used for the first time to characterize the entire faecal microbial population in infants exposed to maternal IAP has shown that the antibiotic prophylaxis has a significant impact on the early fecal microbial composition of infants, which partially recovers after 30 days of life, in particular for some microbial groups such as *Bifidobacterium* spp. Moreover, the initial effect of IAP in term of reduced biodiversity and bifidobacteria counts is greater in BF infants than MF ones. Further studies analysing the vaginal, breast-milk and faecal microbiota from pregnant women and their infants might provide new insights into the effects of IAP exposure.

## Supporting Information

S1 Fig Evaluation of alpha diversity. Estimates of alpha diversity for faecal samples from BF-IAP, BF-C, MF-IAP cohorts at day 30.
(TIF)

S2 Fig Abundance of bacterial families. Relative abundances of bacterial families in BF-IAP, BF-C, MF-IAP and MF-C faecal samples. The Other category contains all other families present at <1% of relative abundance. Black and white colour in print is required.
(TIF)
