# Supplementary figures and images for: Early Gut Microbiota Perturbations Following Intrapartum Antibiotic Prophylaxis to Prevent Group B Streptococcal Disease
Source: PLoS One. 2016 Jun 22;11(6):e0157527. doi: 10.1371/journal.pone.0157527 (PMC4917232; doi:10.1371/journal.pone.0157527)

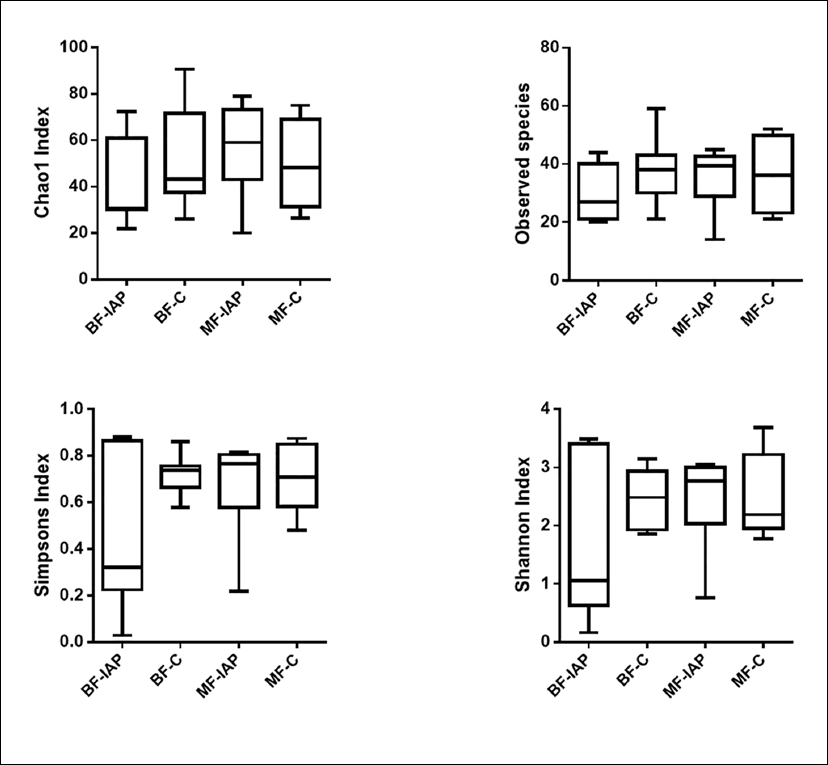

Supplement: S1 Fig — Estimates of alpha diversity for faecal samples from BF-IAP, BF-C, MF-IAP cohorts at day 30. (TIF) [file pone.0157527.s001.tif]

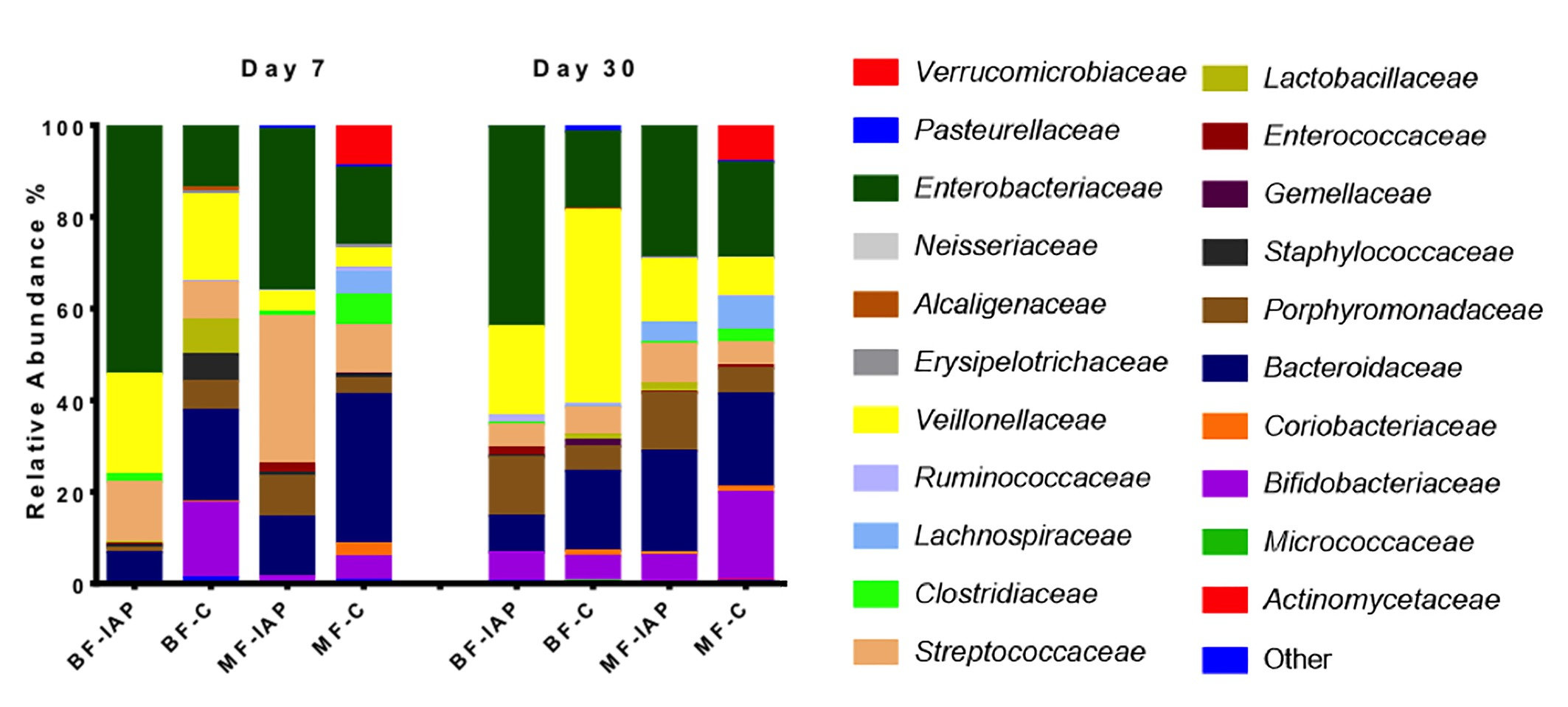

Supplement: S2 Fig — Relative abundances of bacterial families in BF-IAP, BF-C, MF-IAP and MF-C faecal samples. The Other category contains all other families present at <1% of relative abundance. Black and white colour in print is required. (TIF) [file pone.0157527.s002.tif]
